# Identification of a candidate self-incompatibility locus in beet (*Beta vulgaris*)

**DOI:** 10.1007/s00122-026-05368-6

**Published:** 2026-09-23

**Authors:** Gil Yardeni, Thomas Holzweber, Juliane C. Dohm, Heinz Himmelbauer

**Affiliations:** Institute of Computational Biology, Department of Biotechnology and Food Science, BOKU University, Muthgasse 18, 1190 Vienna, Austria

## Abstract

**Key Message:**

This study provides the first genomic evidence for a candidate self-incompatibility locus in beets (*Beta vulgaris*), identifying T2/S-RNase candidate genes and a complex S-locus on beet chromosome 2. A comparison of self-incompatible and self-compatible beet taxa reveals structural degeneration linked to mating system shifts. This work establishes a genomics-first framework for investigating self-incompatibility in understudied taxa and offers important implications for crop breeding and the evolution of plant reproductive systems.

**Abstract:**

The majority of flowering plants maintain various mechanisms to prevent self-fertilization. One such mechanism, the genetic self-incompatibility (SI) system, exists in about half of angiosperms. SI systems have been described for only few taxa, as their diversity and lack of homology renders their study complicated. Yet, an RNase-based SI system (RSI) has emerged as widespread and possibly ancestral to eudicots. RSI has been recognized in at least six plant families and lately also in Caryophyllales. Here, we utilize the accumulated knowledge of RSI systems and high-quality genomic resources to investigate the presence of RSI in beet, *Beta vulgaris.* The genus *Beta* encompasses both self-compatible and self-incompatible taxa. We examined the genes of five beet genome assemblies to identify a candidate for the female component of RSI, a T2/S-RNase, and its corresponding candidate S-locus. A locus on beet chromosome 2 met our criteria, supported by phylogenetics data, sequence characteristics, and expression patterns that are unique to S-RNases. The candidate S-locus in *Beta* was highly repetitive with gene copy number variation observed in different cultivars. By comparing the candidate S-locus of *B. vulgaris* with that of the self-compatible wild beet *B. patula*, we hypothesize a potential link between reproductive modes and S-locus architecture. Our analysis suggests that S-RNase genes derived from an ancestral and highly conserved gene family are present and active in *Beta*, fulfilling a reproductive function. Employing a genomics-first approach, this work contributes insights into T2/S-RNase evolution and lays the groundwork toward elucidating SI in sugar beet and its relatives.

**Supplementary Information:**

The online version contains supplementary material available at https://doi.org/10.1007/s00122-026-05368-6.

## Introduction

The majority of angiosperms produce hermaphroditic flowers bearing both female and male organs, and many are capable of self-fertilization (selfing). Selfing may assure reproduction but bears genetic costs, therefore, most flowering plants reduce or avoid selfing through a wide range of physiological and molecular mechanisms (Barrett 2002; Renner 2014). These include the separation of sexual organs in time (dichogamy) or space (herkogamy), as well as a post-pollination molecular mechanism, termed self-incompatibility (SI), which occurs in an estimated half of all flowering plants (Haring et al. 1990; Fujii et al. 2016; Wang et al. 2021). The SI system operates by recognizing self-pollen through protein-protein interactions and subsequently rejecting the pollen by arresting development. It is commonly governed by two main components, a female and a male determinant, that are linked in a single S-locus and expressed in pistil and pollen, respectively (Haring et al. 1990; Takayama and Isogai 2005).

Changes in reproductive strategies carry profound evolutionary consequences. Selfing rates are theoretically and experimentally associated with loss and gain of genetic variation and with differing paces of diversification and extinction (Charlesworth and Charlesworth 1995; Wright et al. 2013). Self-fertilization rates have been shown to vary within species or populations and are responsive to ecological contexts, with higher selfing rates exhibited at range margins and ecological extremes (Pannell 2015; Encinas-Viso et al. 2020). Self-incompatibility is most widely studied in agricultural species as it bears important consequences for crop improvement. Specifically, identification and characterization of SI-related components is central for manipulating domesticated crops, generation of inbred lines, and implementing plant breeding practices (Muñoz-Sanz et al. 2020; Zhang et al. 2024).

Despite their importance, SI systems have been described in a small number of taxa, as their study is costly, complicated and time-consuming. The molecular components of SI vary tremendously between lineages, indicating that the phenomenon is non-homologous. Rather, it evolved repeatedly in angiosperm history and was lost frequently (Igic et al. 2008; Zhang et al. 2024). In addition to the low homology among individual components, S-loci are highly divergent and polymorphic due to strong negative frequency selection that they experience—meaning they are notoriously difficult to assemble and genotype (Castric and Vekemans 2004; Wright et al. 2013; Genete et al. 2020). It is thus not surprising that SI systems have been described in either economically important crop families like Brassicaceae, Rosaceae, and Papaveraceae, or in families with species bearing large showy flowers, like Cactaceae and some members of Plantaginaceae (Fujii et al. 2019; Li et al. 2019; Ramanauskas and Igić 2021; Vieira et al. 2021; Goring et al. 2023; Zhu et al. 2023). Knowledge about SI systems in wind-pollinated species is especially lacking, seemingly due to difficulties in performing controlled pollination experiments and in collecting material from the typically small, inconspicuous flowers. To our knowledge, SI in a mainly wind-pollinated species was described in the olive family (Saumitou-Laprade et al. 2017; Carré et al. 2021) and in ryegrass (Cornish et al. 1979; Cropano et al. 2021). Notably, while animal-pollinated species commonly possess systems of mixed inbreeding and outcrossing, SI in wind-pollinated plants is often a discrete phenotype (Aide 1986; Barrett 2002; Goodwillie et al. 2005). Research of wind-pollinated plants thus offers predictable mating behavior and insights on underrepresented modes of sexual evolution (Barrett 2002).

In contrast to the diversity of angiosperm plant SI systems, one emerged as particularly common and possibly ancestral among eudicots. Known as RNase-based SI (RSI), this specific mechanism was recognized in at least six plant families, encompassing rosids, asterids and, recently, Caryophyllales (Steinbachs and Holsinger 2002; Ramanauskas and Igić 2021; Lv et al. 2022). RSI is gametophytically-controlled, employing a female and a male determinant. The first is an S-RNase, an endoribonuclease which is member of the family of T2-type RNases (also called T2/S-RNases). T2/S-RNases are known to exhibit a variety of functions; the family is phylogenetically divided into three classes (I, II, and III), and S-RNases associated with SI responses have exclusively been placed within class III. The male determinant is an F-box containing protein, and in the context of SI was termed S-linked F-box (SLF). Variations of the system exist across plant families; yet, the genetic components of all characterized RSIs appear to possess unique features, providing a framework to identify candidate genes in eudicot species with unknown SI systems (Igic and Kohn 2001; Qiao et al. 2004; Kubo et al. 2010; Fujii et al. 2016; Ramanauskas and Igić 2017; Lv et al. 2022). A female SI determinant is expected to be phylogenetically related to other known SI determinants and assigned to class III of T2/S-RNases; it exhibits unique structural features and its expression is limited to the pistil, in contrast to other T2/S-RNases, which are widely expressed across tissues (Ramanauskas and Igić 2017). The male SLF component is accordingly uniquely expressed in pollen and both male and female components are tightly linked in a highly polymorphic locus (Hu et al. 2024; Wu et al. 2024).

The accumulated knowledge of RSI provided the basis for the following work in beets of the genus *Beta*. The subspecies *Beta vulgaris* ssp. *vulgaris* are versatile crops of temperate regions, encompassing four cultivated types, i.e., table beet, leaf beet, fodder beet, and, notably, sugar beet, which is an essential crop for global sugar production apart from sugar cane. *Beta* section *Beta* includes four wild beet taxa, of which sea beet (*B. vulgaris* ssp. *maritima*) is believed to be the progenitor of all beet cultivars (Biancardi et al. 2012). Wild beets are typically adapted to coastal regions, prevalent along the Mediterranean and European Atlantic coasts. The subspecies *B*. *v*. *maritima* is self-incompatible, producing several to hundreds of inconspicuous hermaphroditic wind-pollinated flowers, though occurrences of gynodioecy have been noted (Goldman and Navazio 2008; Dufay et al. 2009; Leys et al. 2014). The wild beet *B. patula*, a rare and critically endangered species, is endemic to Madeira. Its fragmented population consists of fewer than 3000 individuals across two small uninhabited islets (De Carvalho et al. 2010; Nóbrega et al. 2021). Though closely related to sugar beet and sea beet, *B. patula* is self-compatible, inbreeds naturally, and exhibits lower heterozygosity compared to its sister species (De Carvalho et al. 2010). Obligate outcrossing in *Beta* is maintained by mild protandry and gametophytic self-incompatibility with the exception of self-compatible crop lines (Owen 1942; Goldman and Navazio 2008; Leys et al. 2014). Self-compatibility is a highly desirable trait for plant breeders, and sugar beet breeding lines are mainly self-compatible, enabling the preservation of selected qualities through inbreeding (Mackay et al. 1999; McGrath and Panella 2018). Despite the importance of characterizing reproductive mechanisms in sugar beet, the molecular basis of SI in beets is unknown and research on the topic has been sparse in recent decades.

The past 20 years have witnessed a substantial expansion of genomic resources for sugar beet and its wild relatives (Schneider et al. 2007; Dohm et al. 2012, 2014; Sandell et al. 2022). These resources have been instrumental in investigating genetic diversity and the phylogenetic relationships between cultivated varieties and their wild progenitors (Galewski and McGrath 2020; Rodríguez del Río et al. 2019; Sandell et al. 2022; Schmidt et al. 2024) enabling functional investigations of genes critical for cultivation (Pin et al. 2012; Stracke et al. 2014; Capistrano-Gossmann et al. 2017; Lehner et al. 2021; Felkel et al. 2023; Sielemann et al. 2023; Sandell et al. 2025; Dohm et al. 2025). Here, we utilize these comprehensive resources to perform a systematic examination of T2-type RNase genes with the goal of identifying a candidate SI-related S-RNase and its corresponding candidate S-locus in beets. By comparing with self-compatible *B. patula*, we explore the potential link between the SI phenotype and the S-locus architecture.

## Materials and methods

### Genomic and transcriptomic resources

The analysis was performed using four long-read assemblies of sugar beet (Table 1). We used two assemblies of the double-haploid genotype KWS2320, generated with different technologies: RefBeet-1.95 is a preliminary in-house version of RefBeet-3.0 (Dohm et al. 2025) generated using Pacific Biosciences long-read sequencing. Assembly 2320BvONT (Sielemann et al. 2023) based on ONT nanopore technology was downloaded from https://pub.uni-bielefeld.de/record/2966927. We used three additional assemblies, previously constructed from lines EL10 (version EL10.2, McGrath et al. 2022, obtained from https://phytozome-next.jgi.doe.gov/), Strube U2BV v.1.0 (henceforth U2BvONT; Sielemann et al. 2023, https://pub.uni-bielefeld.de/record/2966927), and *B. patula* short-read genome assembly Bpat-1.0 (Rodríguez del Río et al. 2019; https://bvseq.boku.ac.at/Genome/Download/Bpat/). We utilized the existing annotations for all assemblies except RefBeet-1.95 and publicly available RNA-seq data associated with the assemblies (Table S1). The RefBeet-1.95 gene annotation was generated using Augustus v3.5.0 (Stanke et al. 2008) using a Caryophyllales-specific species model (Minoche et al. 2015).

**Table 1:** T2/S-RNases and SLF genes identified in four genome assemblies of sugar beet (*Beta vulgaris* ssp. *vulgaris*). T2 (BLAST): T2/S-RNase genes identified by BLAST in the gene sets of the respective genome assemblies; SLF (BLAST): SLF genes identified by BLAST in the gene sets of the respective genome assemblies; S-locus (bp): Size of the candidate S-locus on beet chromosome 2; RNase genes: Number of T2/S-RNase genes in the candidate S-locus (curated annotation); F-box genes: Number of annotated SLF genes in the candidate S-locus (curated annotation). See also Fig. 4

Assembly	Genotype	T2 (BLAST)	SLF (BLAST)	S-locus (bp)	RNase genes	F-box genes
RefBeet-1.95	KWS2320	25	164	128,076	4	5
2320BvONT	KWS2320	18	206	199,370	6	6
EL10.2	EL10	17	187	71,026	2	3
U2BvONT	U2Bv	15	201	76,776	2	3

### A phylogenomic approach for detecting S-RNases and F-box genes

To detect S-RNase candidate genes, we employed a phylogenomic approach. We used a database of known T2/S-RNase proteins (Ramanauskas and Igić, 2021; downloaded from https://github.com/karolisr/ramanauskas-igic-2021a) and added recently published T2/S-RNases. Protein sequences of T2/S-RNases from Caryophyllales species *Schlumbergera truncata* and *Matucana madisoniorum* were extracted with the kakapo pipeline (Ramanauskas and Igić 2023). Candidate S-RNases for *Selenicereus* spp. were obtained from Li et al. (2022) and translated into protein using transeq (Rice et al. 2000). We performed functional annotation using eggnog-mapper v.2.1.12 (Cantalapiedra et al. 2021) based on eggNOG orthology data (Huerta-Cepas et al. 2019) and retained sequences that were homologous to RNases. We also included genes annotated as T2/S-RNase in *Eucommia elmoides* (PRJNA357336; Qing et al. 2021), *Amaranthus hypochondriacus* v2.1 (from Phytozome; Lightfoot et al. 2017), and *Dianthus caryophyllus* (http://carnation.kazusa.or.jp; Yagi et al. 2014).

The annotated gene sequences of four sugar beet genome assemblies were queried against the database using blastp (Camacho et al. 2009). T2/S-RNases are highly diverged; we retained matches with > = 20% identity, a subject sequence length of > = 50 amino acids and an *e*-value < 1e^−20^. In addition to previously published functional annotation for each assembly, we annotated the detected candidates using eggnog (see above) and a HMMER search (Eddy 2011). The retrieved candidates, including all annotated gene transcripts (see Data S1 and Data S2 for recovered RefBeet sequences), were added to the T2/S-RNase database and were aligned using mafft (Katoh and Standley 2013) with accurate and sensitive alignment, running as --ep0 --genepair --maxiterate 1000. A T2/S-RNase phylogenetic tree was obtained for the total of 691 sequences with iqtree2 v.2.2.0 (Nguyen et al. 2015) using ModelFinder to select the optimal model of sequence evolution (Kalyaanamoorthy et al. 2017) and assessing branch support with 1000 replicates for ultrafast bootstrap approximation. IQTREE2’s ModelFinder inferred WAG + R8 as the best-fit substitution model. After retrieving a phylogeny, T2/S-RNases assigned to class III were extracted using the get_taxa_name() function in the ggtree R package (Yu et al. 2017) and aligned to construct a T2/S-RNase gene tree as described above.

To obtain a phylogenetic tree of candidate SLF genes, we obtained a database of known F-box and SLF/FBA-domain-containing genes (Ramanauskas and Igić 2021; https://github.com/karolisr/ramanauskas-igic-2021a). We added candidate SLF/FBA genes of *Schlumbergera truncata* (Ramanauskas and Igić 2021), *Selenicereus* spp. (Li et al. 2022) and *Rosa chinensis* and *R. multiflora* (Vieira et al. 2021) as described above. The latter two were translated and functionally annotated (see above). Filtering blastp results was as above, except that only subject sequences with length of > = 100 amino acids were kept (see Data S1 and Data S2 for recovered RefBeet sequences). The sequences were aligned and a tree was constructed as above. IQTREE2’s ModelFinder inferred JTT + F + R8 as the best-fit substitution model.

All trees were plotted and explored with the R packages ggtree (Yu 2020), treeio (Wang et al. 2020), and ape (Paradis et al. 2004).

### Investigating protein features and expression typical to S-RNases

Functional S-RNases display unique features, providing a roadmap to discern likely candidates in sugar beet. We expect S-RNases to display a strongly basic pI, to contain two introns and two highly conserved histidine residues (Roalson and McCubbin 2003; Ramanauskas and Igić 2017, 2021). We calculated the pI of all candidate proteins with the Bio.SeqUtils.IsoelectricPoint in biopython v.1.83 (Cock et al. 2009) and confirmed the annotated gene models by assembling the gene transcripts with StringTie v.2.2.1 (Pertea et al. 2016), using public RNA-seq data (Table S1). RNA-seq data were available from root and leaf tissues of U2BvONT and from root, leaf, seedling, seed, and inflorescence tissues of KWS2320. No RNA-seq data were available for EL10, but we used data sequenced from root and leaf tissues of its progenitor genotype C869 and from seeds of its sibling EL-A027008 (Table S1). All RNA reads were first corrected for sequencing errors using Rcorrector v.1.0.5 with default settings (Song and Florea 2015) and quality-filtered with Trimmomatic v.0.39 (Bolger et al. 2014) with the settings ILLUMINACLIP:TruSeq3-PE.fa:2:30:10:2:True LEADING:3 TRAILING:3 MINLEN:35. The reads were aligned with STAR 2.7.10b (Dobin et al. 2013) against genomic sequences with parameters --quantMode TranscriptomeSAM --outFilterMismatchNmax 4 --seedSearchStartLmax 10 --alignIntronMax 2000 --alignMatesGapMax 2000 --peOverlapNbasesMin 10 --twopassMode Basic --outSAMstrandField intronMotif. Each RNA-seq dataset was aligned to its isogenic assembly; data derived from KWS2320 were aligned to RefBeet.

To investigate the expression of candidate S-RNase genes in different tissues, we performed transcript quantification with RSEM (Li and Dewey 2011), suitable for the task with its statistical modeling of mapping uncertainty. Expression was calculated using default settings, followed by the script rsem-generate-data-matrix to combine any single-sample results into a unified matrix. For comparative quantification of expression in different tissues, we used data sampled from KWS2320, as inflorescence was not sampled from other genotypes. We additionally confirmed the expression or lack thereof of candidate genes in the leaf and root of all other genotypes. TMM-normalized expression counts (cpm; Robinson and Oshlack 2010) were calculated with edgeR v.4.0.16 (Robinson et al. 2010) in R v.4.3.3 (R core team 2024), by calculating normalization factors with the calcNormFactors() function and extracting cpm values with cpm().

### Collinearity of a putative S-locus in sugar beet and in *B. patula*

We investigated the structure of a putative S-locus on BvChr2 in four sugar beet assemblies and in *B. patula*. We anchored the search on genic regions flanking the putative S-locus. Each flanking region included two anchor genes that were similarly annotated in at least three assemblies, i.e., a PEX-1N AAA-ATPase complex gene and a spindle and kinetochore-associated protein 2 (*SKA2*) on the 5’ end and a 3-oxoacyl-[acyl-carrier-protein] synthase III gene and a G-patch/zinc finger CCCH domain-containing protein in the 3’ flanking region (Table S2). We considered as candidate S-loci the genomic coordinates between the anchor genes where a candidate S-RNase was present.

Each assembly was mapped against RefBeet with minimap2 v.2.26 (Li 2018) using the parameters -cx asm20 in order to perform intraspecific mapping allowing up to 20% sequence divergence. Adjacent alignments were merged and blocks were visually inspected with asynt (https://github.com/simonhmartin/asynt, v 0.1). In *B. patula*, we identified the candidate S-locus based on synteny shared with sugar beet and gene orthology (Rodríguez del Río et al. 2019).

We used spaln v.3.0.2 (Gotoh 2008) to perform splice-aware mapping of the RefBeet protein sequences against each genome assembly, limiting the search to genes on the putative S-locus and its flanking regions, with settings -S3 -O0,1,2,5,7 -T ../Eudicoty -LS -XQ99 -M1 -N 200 -XS, using alignment parameters specific to eudicots and controlling the intron length distribution and researching the query region not aligned in the first trial. All positively scored blocks were examined (“salvage mode”) and we increased the Point Accepted Mutation (PAM) score to 200, in order to allow mapping of sequences diverged by up to 70%. Finally, we compared the nucleotide regions mapped by spaln to those in RefBeet to identify unannotated putative RNases and F-box genes. We assumed conserved gene order between genotypes; when several syntenic blocks were found, we selected the “true” block according to gene order. When coordinates of regions did not match, the best syntenic block was chosen by the similarity score of the alignment.

### Computing resources and data management

Data were analyzed on a Linux cluster with nodes of up to 64 cores and a maximum of 1 TB RAM. Further analyses were run on VSC-5 nodes of the Austrian Scientific Computing Infrastructure (up to 128 cores and 1 TB RAM). Scripting was performed using Perl 5 and Python 3. Standard Linux tools were used for data handling.

## Results

### Phylogenomic analysis highlights the dynamic nature of T2/S-RNase genes

To identify candidate genes for S-RNase proteins in beets, we searched for T2-type RNase homologues in four annotated sugar beet genome assemblies representing three different genotypes (McGrath et al. 2022; Sielemann et al. 2023; Dohm et al. 2025) and proceeded to refine the search for candidates according to their phylogenetic placements, sequence characteristics, and expression patterns unique to S-RNases. We constructed a database of 615 known T2/S-RNases including protein sequences from 186 plant species and used blastp to query it against the annotated protein sequences derived from the four sugar beet genome assemblies (Table 1). We identified between 15 and 25 candidate genes in each assembly (Transcripts counted separately; Table 1; Table S3), added them to the database, and constructed a T2/S-RNase protein sequence phylogeny.

**Fig. 1:**
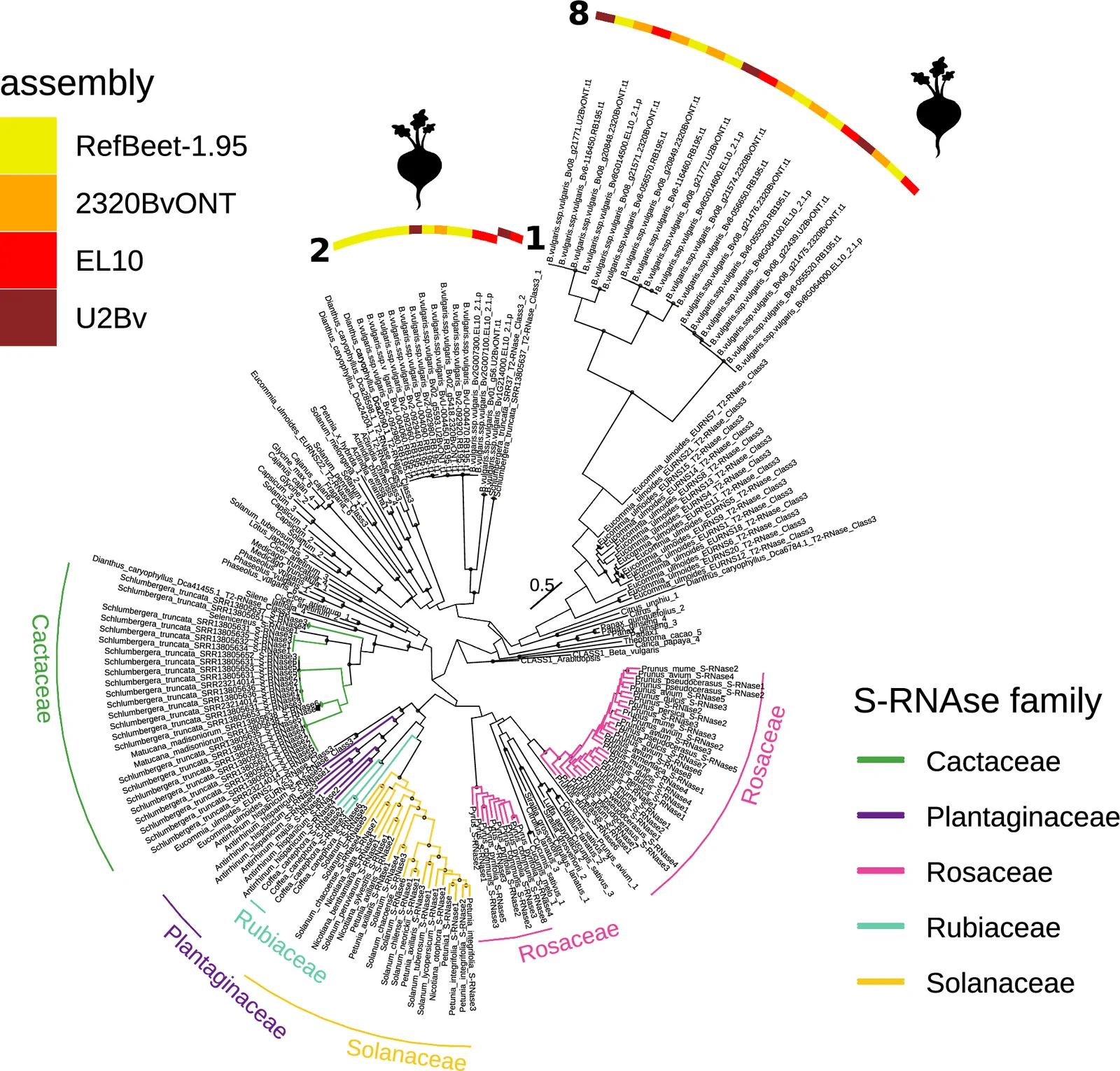
Maximum-likelihood phylogeny based on protein sequence alignments of class III T2/S-RNase genes including S-RNase candidate genes identified in four different sugar beet genome assemblies representing three different genotypes (Table 1). Assemblies RefBeet and 2320BvONT are both derived from the sugar beet reference genotype KWS2320. Proteins with known S-RNase function are marked by color and taxon family name

Based on their sequence, T2/S-RNase genes can be assigned to one of three classes I, II, or III. In previous studies, dicot T2/S-RNase genes involved in an SI response (S-RNases) were consistently assigned to class III. A lack of class III T2/S-RNases in a species is commonly indicative for the lack of an RNase-based SI mechanism (Igic and Kohn 2001; Zhao et al. 2022). In addition, S-RNases from different taxa are often, but not necessarily, closely related (Honsho et al. 2021; Lv et al. 2022). Our phylogeny recovered three well-supported T2/S-RNase subgroups corresponding to classes I to III (Fig. S1). While each class was highly supported, relationships within each subgroup remained widely unresolved, likely due to the high pairwise divergence within this ancient gene family. Within class III T2/S-RNases (Fig. 1), known S-RNases clustered according to plant families and did not form a monophyletic group, as a few additional T2/S-RNases of unknown SI function were interspersed within. The immediate relatedness of T2/S-RNases is marginally informative of their function, as saturation due to high substitution rates and homoplasy are both expected (Luhtala and Parker 2010; Lv et al. 2022).

**Fig. 2:**
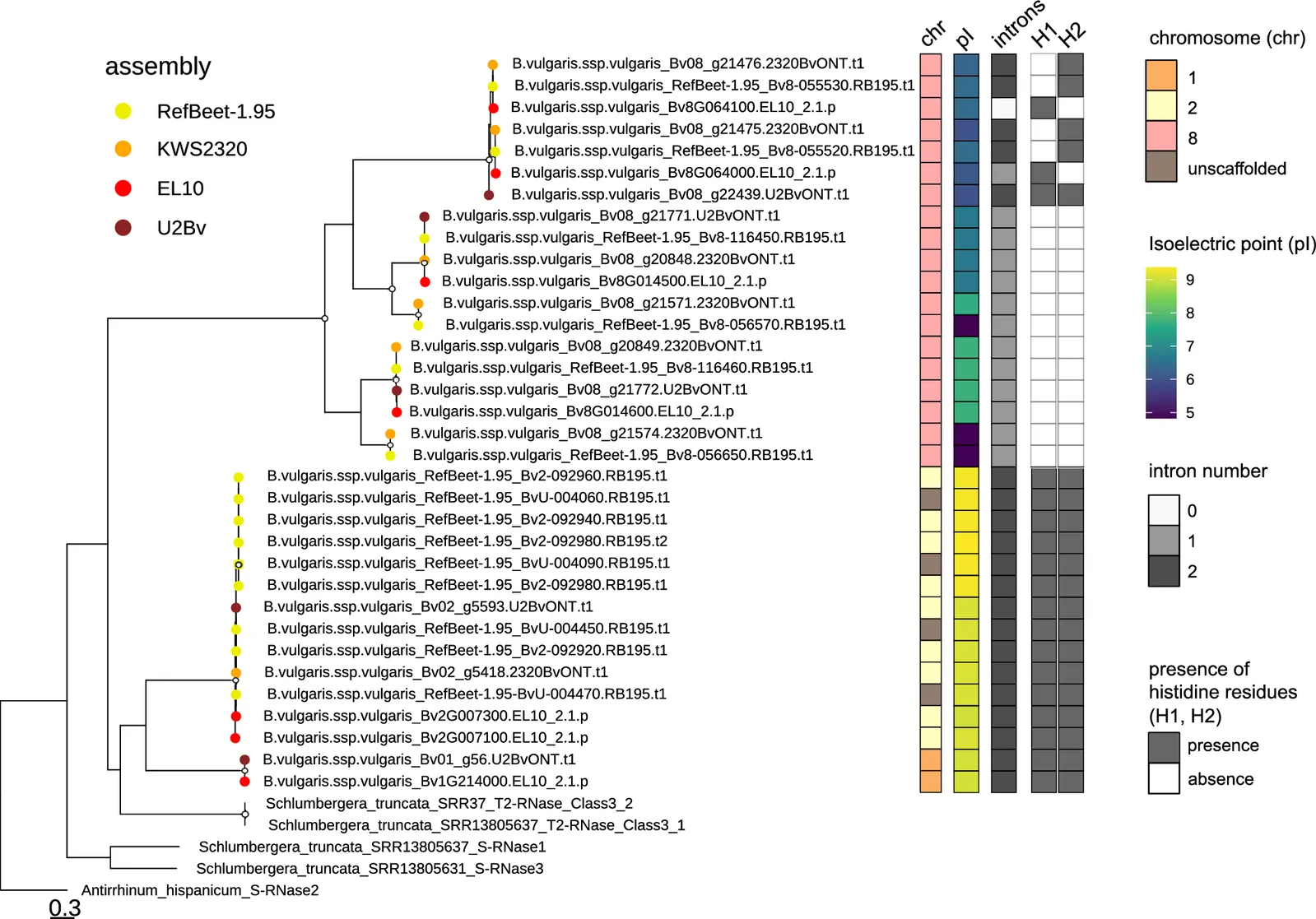
Maximum-likelihood phylogeny of protein sequences of S-RNase candidate genes in sugar beet. Nodes with bootstrap support above 95% are marked by a circle. Colored circles indicate the source genomic assembly of the gene, whereby KWS2320 corresponds to assembly 2320BvONT. Gene/protein properties are displayed in adjacent bars at the right-hand side. Chr: chromosomal localization of the gene; pI: isoelectric point of the predicted protein; introns: intron number; H1, H2: presence/absence of conserved histidine residues. Functional S-RNases are characterized by a strongly basic pI, two introns and two conserved histidine residues (Ramanauskas and Igić, 2017)

Identified T2/S-RNase genes from sugar beet clustered into nine groups (Fig. S1), two of which were assigned to class III (Fig. 1) and thus candidates for possessing SI function. Of the two groups assigned to class III, one included genes located on chromosome 8 (BvChr8) while the second contained genes on BvChr1 and BvChr2—consistently for all four beet genome assemblies. Four class III T2/S-RNase genes, unique to the RefBeet-1.95 assembly (henceforth RefBeet), were located on two unscaffolded contigs. None of the sugar beet subgroups assigned to class III were retrieved as closely related to known S-RNases-instead, one group was closely related to putative non-S-RNases of the false Christmas cactus (*Schlumbergera truncata*; Figs. 1 and 2). While genes assigned to classes I and II contained 1:1 pairwise orthologs between assemblies, the clusters assigned to class III included varying numbers of genes from each assembly ranging from 15 (in RefBeet) to five (in U2BvONT; Fig. 2) indicating either functional differences in SI systems and/or differences due to technical reasons (assemblies, gene annotation). We also noticed that *Selenicereus* spp. T2/S-RNase genes were assigned to various phylogenomic classes (Fig. S2), suggesting that the resource of Li et al. (2022) contained both S-RNase and other T2/S-RNases.

In RSI systems, male SLF genes can similarly be classified by phylogenomic placement and sequence properties. This phylogeny is generally less informative in the search for candidate genes involved in SI, as SLF genes are considered young and fast-evolving (Lv et al. 2022; Zhu et al. 2023). We used the same sequence-based approach as above to search for SLF genes with a collection of 542 protein sequences of known SLFs. We identified between 164 and 206 SLF gene candidates in each sugar beet genome assembly (Transcripts counted separately; Table 1, Table S4), which were distributed over all chromosomes. In the phylogenetic tree, sugar beet SLF candidates formed several large clusters (Fig. S3). Three groups were moderately related to Rosaceae SLF genes and one was related to *Schlumbergera* candidate SLFs. We found no immediate candidates for a sugar beet SLF, likely due to the large number of F-box proteins in the sugar beet genome.

### Gene properties and expression identify S-RNase candidates

We examined the identified sugar beet T2/S-RNase genes assigned to class III for the properties expected of functional S-RNases, i.e., a strongly basic isoelectric point (pI), a gene structure containing two introns, and two highly conserved histidine residues at the RNase active site (Igic and Kohn 2001; Ramanauskas and Igić 2017; Lv et al. 2022). Of the two clusters assigned to class III, only one showed the above characteristics (Fig. 2; Table S3; Fig. S5). The pI values of all beet candidate S-RNases exhibited a marginally bimodal distribution and a distinct group had pI values > 8 (Fig. S5), typical for S-RNase and S-like RNase genes. The candidates’ exonic gene lengths ranged from 797 to 1558 bp with a coding region of 549–723 bp, similar to Cactaceae S-RNase gene structures (Ramanauskas and Igić 2021). These putative S-RNase genes were located at various genomic regions: a total of nine genes in all genome assemblies were located on BvChr2 and four genes were located on two unscaffolded RefBeet contigs. Additionally, one candidate gene each in EL10 and U2Bv, respectively, were located on BvChr1 (Fig. 2).

We next used publicly available RNA-seq data matching the de novo sequenced beet genotypes to confirm the expression of the candidate genes and their intron-exon structure. We found that the genes located on BvChr2 and unscaffolded contigs were almost exclusively expressed in inflorescence tissue with the exception of candidates on BvChr1 (see below). We confirmed the intron-exon junctions of T2/S-RNase genes annotated on BvChr2 in RefBeet based on isogenic inflorescence RNA-seq data; yet, the high collinearity between genotypes allowed us to assume that transcript structure is conserved within the subspecies. For one of these genes two transcripts were annotated but only the expressed version *Bv2-092980.RB195.t1* was kept for further analysis (Table S3).

The pairwise amino acid sequence identity of S-RNase candidates among different genotypes ranged between 89.1 and 98.7% (Fig. S6), differing from the expectation of high polymorphism and low identity between T2/S-RNases. In comparison, identity between T2/S-RNases of *S. truncata* ranges from 50.5 to 70.1% (Ramanauskas and Igić 2021). In an intraspecific comparison, the candidates shared a nearly similar sequence identity-average 21%-with both *S*. *truncata* S-RNases and non-S-RNases. A protein sequence comparison to *S. truncata* S-RNase revealed the presence of two highly conserved histidine residues (Fig. S7), and a gene-level comparison indicated a conserved exon-intron structure. Candidates on BvChr1 presented a slight variation in intron position.

**Fig. 3:**
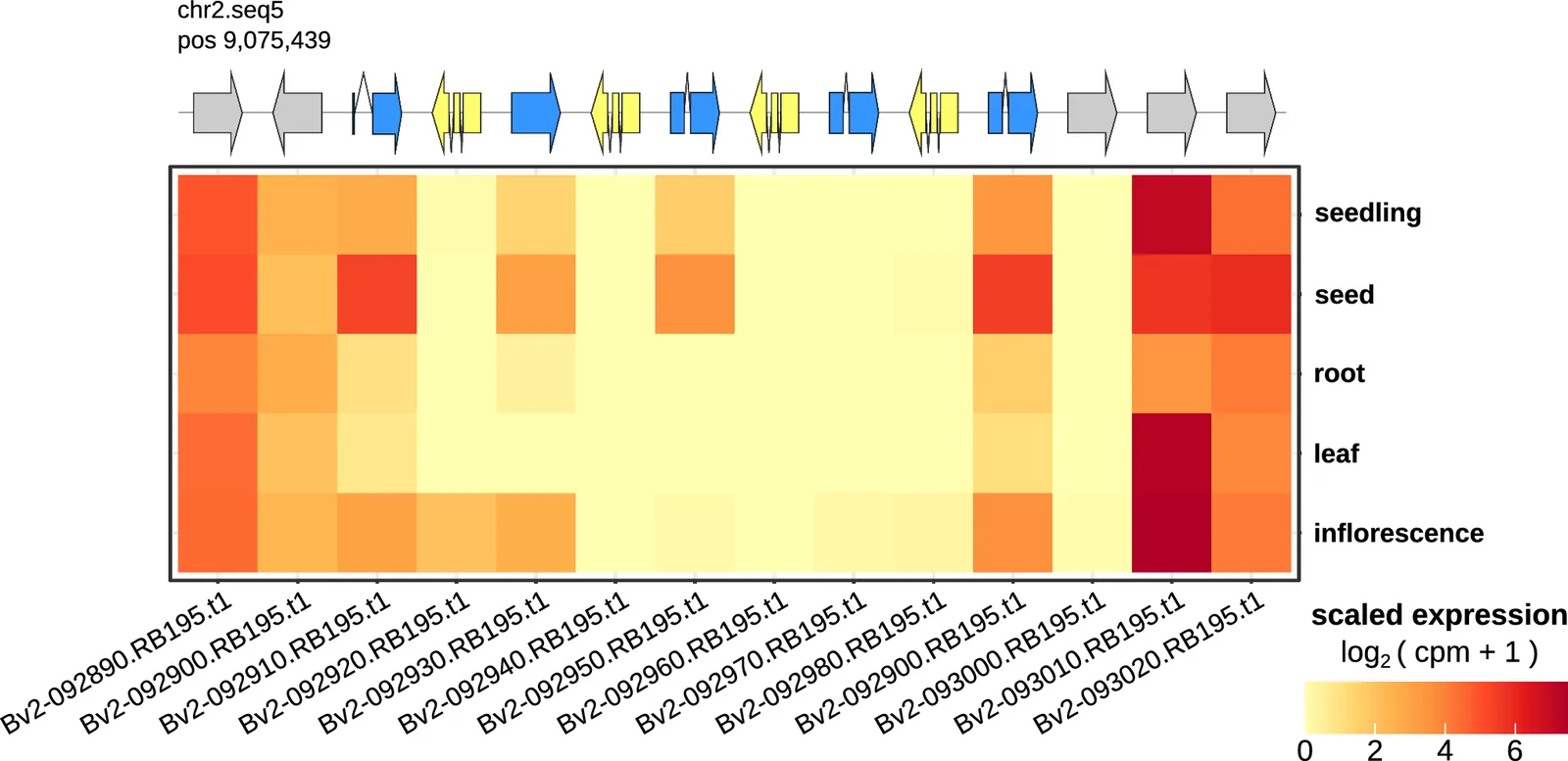
Structure of a candidate S-locus on beet chromosome 2 based on the RefBeet assembly and expression of the genes encoded therein. Genes in yellow color: putative S-RNases; blue: F-box genes; gray: anchor genes unrelated to SI. For gene expression in five different tissues, median normalized transformed expression values were calculated

A female component in an RSI system should be expressed exclusively in pistil. Hence, its expression in other tissues such as leaf, root, seed, or seedling would not be expected. We examined the gene expression patterns of all beet candidate S-RNase genes and found two RefBeet genes exclusively expressed in inflorescence tissue, i.e., *Bv2-092920.RB195* on BvChr2 (Fig. 3) and *BvU-004470.RB195* on an unscaffolded contig (Fig. S8). The scaled expression was found in one of two inflorescence samples at low yet detectable range of 3.74–4.05 log-CPM (counts per million). Other S-RNase candidates were expressed in inflorescence with low values between 0.04 and 0.62 log-CPM and one was expressed with 0.12–0.17 log-CPM in seed and root. We found a low level of expression of *Bv1G214000.EL10_2.1*, the candidate on BvChr1 in the sugar beet genotype C869 leaf and root (range 0.07–0.19 log-CPM). The probable ortholog of the gene in U2Bv, *Bv01_g56.U2BvONT.t1*, was also expressed in root (0.12–0.6 log-CPM). In summary, candidate genes on BvChr1 are less likely to participate in SI response.

### A complex putative S-region in beets exhibits gene duplication

**Fig. 4:**
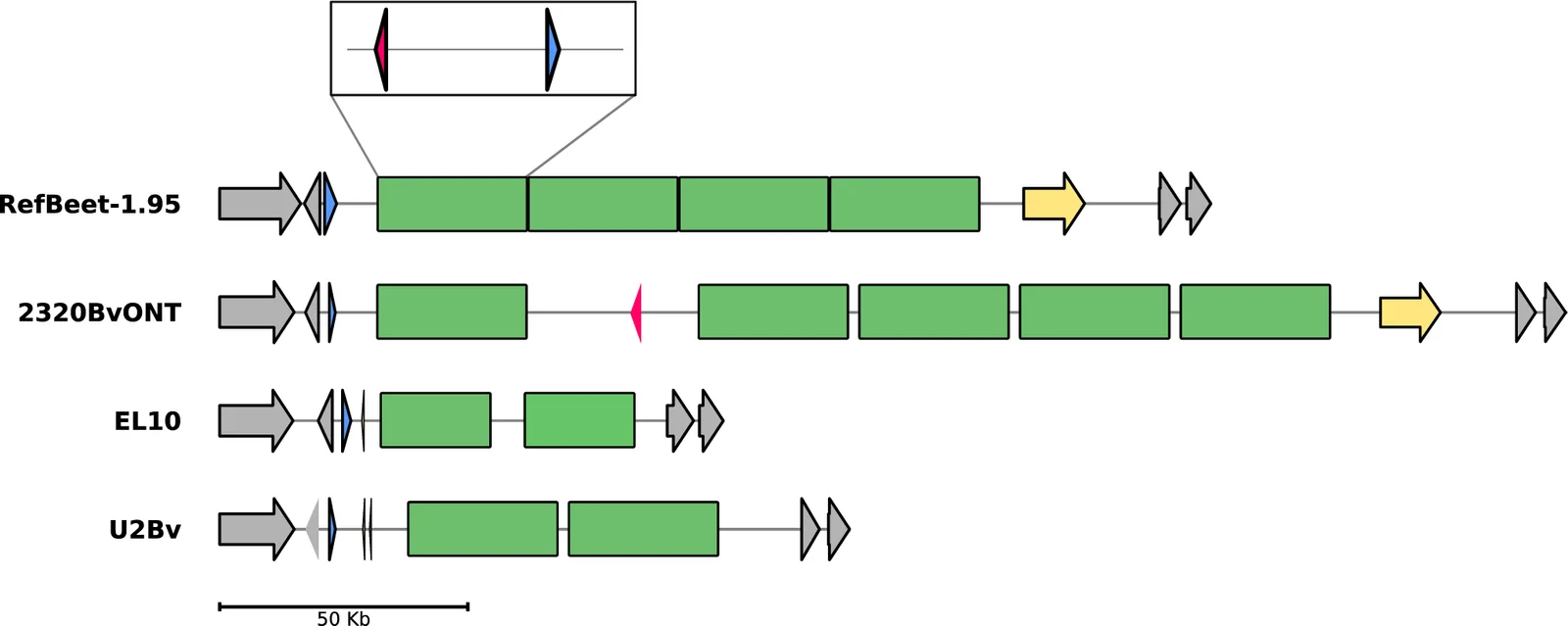
Comparison of a putative S-locus in four sugar beet genome assemblies. Blue arrows: F-box genes; pink: T2/S-RNase; gray: conserved anchor genes unrelated to SI; yellow: anchor genes annotated in some, but missing from other assemblies; green blocks: pairs of one F-box gene and one T2/S-RNase. Arrows lacking black borders: absent from the original annotation but identified during mapping

Sequence properties and gene expression in different tissues pointed to four S-RNase sugar beet candidate genes, all populating a single region on BvChr2. In RefBeet, the locus (henceforth: candidate S-locus) spans 128,076 bp and displays an architecture in which each T2/S-RNase gene is flanked by two genes annotated as F-box genes (Fig. 4). Of the five F-box genes, all were expressed in various tissues (Fig. 3). A single F-box gene (*Bv2-092970.RB195.t1*) was expressed at very low levels in inflorescence (0.44 log-CPM) and in root (0.66 log-CPM). The two unscaffolded contigs in RefBeet that contained S-RNase candidates were 44,964 bp and 52,879 bp in length and contained four and three genes, respectively. All genes on the two short contigs were annotated as RNases or as F-box containing genes except for a single gene, annotated as a carbon-nitrogen hydrolase.

We found differences in the number of genes within the candidate S-locus in individual beet genome assemblies-four T2/S-RNase genes were identified in RefBeet, and in contrast six such genes in 2320BvONT (five in the original annotation; one further gene identified on closer inspection; Table S5), despite the two originating from the same genotype (Table 1). These discrepancies are indicative of either a highly dynamic and recent duplication, mis-assemblies or mis-annotation, all of them ubiquitous phenomena regarding S-loci. The putative S-locus of RefBeet contained two groups of T2/S-RNase genes, displaying different characteristics (Fig. S6). The first group was observed as orthologous between assemblies in varying copy numbers (*Bv2-092920.RB195.t1* on BvChr2 and *BvU-004450.RB195.t1* on an unscaffolded contig) and the second included genes present in several copies exclusively in RefBeet, which showed little to no expression in the sampled tissues (*Bv2-092940.RB195.t1*, *Bv2-092960.RB195.t1*, *Bv2-092980.RB195.t1* on BvChr2 and *BvU-004060.RB195.t1*, *BvU-004090.RB195.t1* on unscaffolded contigs). The two groups exhibited differences in genomic sequence. Most notably, the genes of the second group contained a 13 bp deletion at the start of exon 2, leading to a frameshift affecting nine amino acids; a 4 bp insertion restored the disrupted open reading frame (Fig. S7).

### The candidate S-locus is collinear in sugar beet, degraded in *B. patula*

We next sought to investigate collinearity of the putative S-loci between genome assemblies and species, particularly with the self-compatible wild beet *B. patula.* The candidate regions were difficult to compare directly, due to mis-annotations and a highly repetitive gene content. However, the regions flanking the putative S-locus were highly conserved, providing a baseline to the comparison. We considered two common flanking anchor genes at each side of the putative S-locus, i.e. genes *Bv2-092890.RB195.t1* and *Bv2-092900.RB195.t1* (upstream) *Bv2-093010.RB195.t1* and *Bv2-093020.RB195.t1* (downstream; Fig. 3, Table S2). We identified putative S-loci with varying length in the other sugar beet genome assemblies, ranging from 71,026 bp in EL10 to 199,370 bp in 2320BvONT (Table 1; Fig. 4; Table S2; Table S5). The regions had previously been functionally annotated with varying numbers of T2/S-RNase genes, ranging from two in EL10 and U2BvONT to five in 2320BvONT. Each T2/S-RNase gene was flanked by an F-box-containing gene, with numbers ranging from three in EL10 and U2BvONT to seven in 2320BvONT. The annotated T2/S-RNase genes were often shorter than 400 bp (in contrast to approx. 1350 bp in RefBeet) while some F-box genes were as long as 3244 bp (RefBeet: 1577–2440 bp), suggesting that some genes may be truncated, fused or mis-annotated. In a comparison of the candidate S-loci between assemblies, the annotated F-box genes exhibited pairwise amino acid sequence identity of 86.6–100%. To further investigate the presence of structural variation (SV) or genic regions missing from annotation, we performed protein-to-nucleotide splice-aware mapping. The comparison between S-loci indicated SVs between genotypes, and unannotated genes may be present in some genotypes (Fig. 4; Table S2; Table S5). Mapping of RefBeet protein sequences onto the other beet genome assemblies confirmed the functional annotation of most genes as well as high identity of the coding regions. It again indicated that additional unannotated genes may be present. Specifically, we found an unannotated region in 2320BvONT that mapped to a RefBeet T2/S-RNase. Interestingly, gene content also differed in genic regions spanning the candidate S-locus: a 12 kbp genic region with Pfam annotation DUF4371 was present in both KWS2320 assemblies but absent in others. Two genic regions of unknown function were present in U2BvONT, but only one was annotated in EL10-and both were absent from KWS2320 genotypes (Fig. 4).

**Fig. 5:**
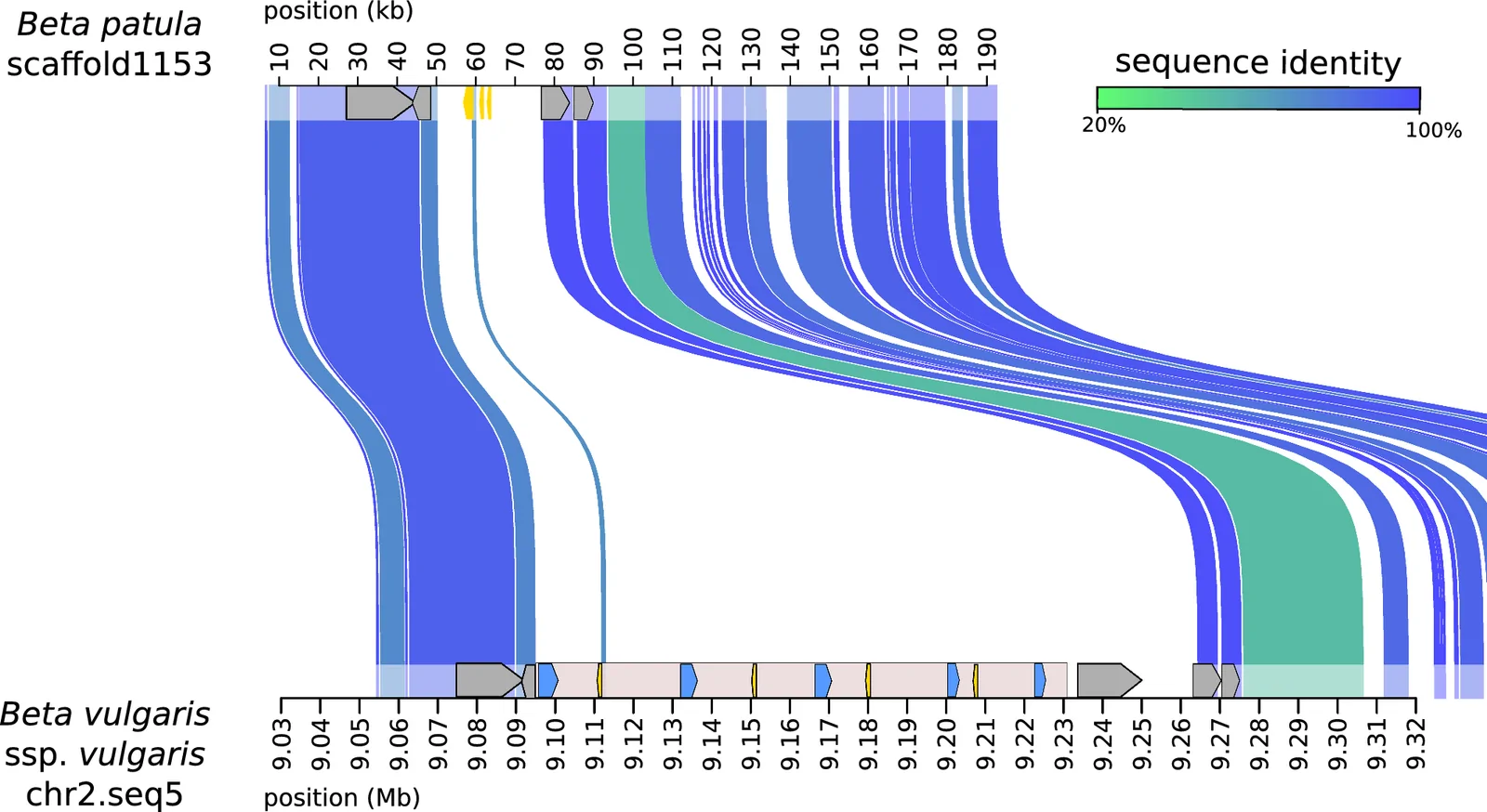
Comparison of the structure of a putative S-locus between the wild beet *B. patula* (top) and sugar beet (bottom), including flanking regions. Colored lines indicate the average sequence identity of each block. Gray arrows: anchor genes unrelated to SI; yellow: T2/S-RNase; blue: F-box genes. Yellow arrows lacking outline in *B. patula* represent RNase genes or gene fragments newly identified by mapping (not annotated in *B. patula* genome assembly Bpat-1.0)

In the genome assembly of the self-compatible wild beet *B. patula* (Rodríguez del Río et al. 2019), the putative S-locus was located on scaffold 1153 (length 497 kbp), spanning fewer than 30 kbp, less than half the size of the shortest putative S-locus in sugar beet. Scaffold 1153 was highly collinear to a 490 kbp region of BvChr2 in sugar beet, with an overall average of 84.3% sequence identity and an average sequence identity of 92.6% at the regions flanking the putative S-locus. Enclosed by the flanking regions, the sequences of the putative *B. patula* S-locus appeared to be divergent and could not be reliably aligned to RefBeet, with the exception of a short 998 bp intergenic block (1220 bp in RefBeet; Fig. 5). A protein-to-nucleotide alignment confirmed both the high conservation of flanking genes and the extreme divergence of the putative S-region. Sequence identity of flanking genes ranged from 76.9 to 99.3%, while the protein sequences of all putative S-RNase genes aligned to a single genomic region in *B. patula* with identities ranging from 57.2 to 58.7%. The alignment contained two frameshifts–caused by a nucleotide insertion and a deletion–as well as a comparatively large intron of 4680 bp (more than four times the average intron length in *B. patula*; Rodríguez del Río et al. 2019), indicating that the *B. patula* S-locus sequence has possibly undergone rearrangements. Moreover, the retrieved RNase sequence in *B. patula* contained numerous substitutions in otherwise highly conserved regions in T2/S-RNase genes (Fig. S9); hence, the region likely contains a non-functional pseudogene.

## Discussion

Self-incompatibility in sugar beet was last comprehensively studied in the 1970s. Observations at the time suggested that obligate outcrossing is mediated by multiple S-loci, a hypothesis that was driven, perhaps, by the complex and non-discrete phenotype in controlled crosses (Lundqvist et al. 1973; Larsen 1977). Contemporary molecular research enables an alternative approach that leverages genomic resources prior to extensive phenotyping, inviting the systematic characterization of homologous systems. Our study is grounded in the widely accepted hypothesis of homology between RSI systems across eudicots and is particularly informed by recent discoveries of an RSI system in Cactaceae (Ramanauskas & Igić, 2017). Despite an approximate 100 million years of divergence between Cactaceae and Amaranthaceae (Yao et al. 2019), our analyses revealed sufficient homology of T2/S-RNase genes to suggest that an ancestral T2/S-RNase operates in sugar beet. We further hypothesize that the homologous candidate genes are involved in reproductive function and may play a role in an SI mechanism similar to those found in rosids and asterids.

*Beta* section *Beta* includes self-compatible and self-incompatible species in a mosaic of breeding phenotypes, a phenotypic diversity also seen in other taxa (De Nettancourt 1997; Ferrer and Good-Avila 2007). The recent loss of SI provides opportunities to juxtapose homologous regions in order to link phenotype and genotype by a straightforward comparison. The lack of homology we found between the closely related sugar beet and *B. patula* highlights the characterized candidate S-region as rapidly evolving, in contrast to an otherwise preserved flanking gene content. The observed decrease in *B. patula* S-locus size and mutations within an encoded T2/S-RNase gene also affecting otherwise conserved amino acid residues, indicate a possible loss of function of this gene. If the locus is indeed involved in an SI response, then rapid change is expected following a phenotypic shift toward self-compatibility.

The genes identified as candidates fulfilled some of the expectations for typical S-RNases. Phylogenetically, the candidate genes clustered in class III as expected; however, they did not form a monophyly with known S-RNases. A monophyly of S-RNase genes was occasionally reported (Ramanauskas and Igić 2021), but not in all examples (Nowak et al. 2011; Ramanauskas and Igić 2017; Zhao et al. 2022), as homoplasy in this ancient gene family may limit the power of inference (Luhtala and Parker 2010; Lv et al. 2022). Our sampling of several Caryophyllales genomes offers some insight into the evolution of T2-type RNases: we note that both groups of sugar beet class III T2/S-RNase genes had close relatives in *Dianthus caryophyllus*; yet, no T2/S-RNase in *Amaranthus hypochondriacus* had been assigned to class III. While the reproductive system of the first is yet to be characterized (Balao et al. 2024), the latter is considered a frequently selfing species (Agong and Ayiecho 1991). It has been previously reported that genera lacking RSI systems will encode no class III T2/S-RNases, as in the case of Brassicaceae (Zhao et al. 2022). Tissue-specific gene expression further contradicted the common expectation from an S-locus. While gene expression patterns for the candidate beet S-RNase were limited to inflorescence, we found a near-constitutive expression of the F-box-containing genes in various tissues. To our knowledge, there are no known SLF genes with pleiotropic function. However, we note that at least one candidate SLF gene was also expressed in other tissues in Cactaceae (Ramanauskas and Igić, 2021).

The interpretation of these findings is limited both by the available data and the tandemly repeated gene architecture. First, sampling of pollen is necessary to pinpoint the role of a gene as a male component. Second, erroneous mapping of reads to the region cannot be excluded, as these may conceal pollen-specific expression, or lack thereof in one or several of the F-box genes. Finally, S-loci are always found to be heteromorphic-hence, the lack of polymorphism between genotypes contradicts our expectation. In beets, the putative S-region includes repeated units spanning 25–50 kbp, a challenging assembly task even for contemporary long-read technology. Hence, the repeated structure may mask heterozygosity. Furthermore, additional alleles may be present on unscaffolded contigs, as the ones we found for RefBeet. Further research on the S-locus in *Beta* will benefit from including wild populations, especially since the processes to construct breeding lines are not entirely transparent and their genetic effects can be unpredictable. We utilized genome assemblies of self-compatible sugar beet cultivars, either double-haploid or inbred-beneficial due to high homozygosity-with the assumption that the main component of self-incompatibility will remain present. The assumption is plausible since transitions from SI to SC were often reported to involve mutations of promoter regions, pollen components (Li et al. 2016) and non-S factors (Wu et al. 2011, 2013; Jiménez-Durán et al. 2013). Moreover, in the sugar beet lines used here, SI is considered to be not the product of degeneration at the S-locus itself, but rather of a single dominant, unlinked self-fertility gene, *Sf*. While not yet characterized, *Sf* is thought to act epistatically to override SI (Owen 1942; Arnaud, 2010). Hence, self-incompatible lines derived from recent breeding efforts are not expected to have lost the S-locus itself.

Repeated genomic elements and gene duplications are recurrent in S-loci and drive loss and regain of SI function. A large number of SLF genes is thought to have proliferated from repeated duplication in both *Antirrhinum* and *Petunia* (Kubo et al. 2015; Li et al. 2019; Zhu et al. 2023). The S-loci in Brassicaceae are also arranged in repeats, indicating a dynamic locus that underwent duplication (Kusaba et al. 2001; Xing et al. 2013). Our findings in sugar beet are the first to describe a similar locus containing not only several F-box-containing proteins, but several copies of T2/S-RNase genes. Since adjacent genes were highly homologous and gene numbers differed between genotypes, duplications in the region may have been very recent followed by loss of function of duplicated genes. In our results, three of the four T2/S-RNase genes acquired an insertion leading to a frameshift (Fig. S7), coupled with low to zero expression in inflorescence. This pattern mirrors a dynamic gene loss and gain similar as described in other S-loci.

In future research, validation of the candidate S-locus and its components will require several complementary lines of evidence. First, tissue-specific sampling of pollen and pistil is needed to directly test the expression and localization of the candidate male- and female-determinant genes, since data used here cannot resolve which components act in which reproductive tissue. Second, extending sampling to closely related genera and to self-incompatible cultivars would help establish whether the candidate S-RNase and F-box genes show the polymorphism and heteromorphism expected of a functional S-locus. Our reliance on self-compatible, highly homozygous genomes may have obscured allelic diversity that is only preserved in outcrossing or SI-retaining lineages. Finally, controlled crosses paired with transcriptomic profiling, following the approach of Ramanauskas and Igić (2021), would allow segregating S-genotypes to be linked directly to phenotypes.

The T2/S-RNase gene family is both ancient and widespread, being present in nearly all organisms and serving a variety of biological functions (Luhtala and Parker 2010; MacIntosh 2011; Rojas et al. 2018). In plants, the family underwent expansion and recent phylogenies suggest that classes I and III arose from the core eudicot gamma genome triplication event (Lv et al. 2022; Liu 2025). The phylogenomic assignment and molecular properties of T2/S-RNase genes are informative in the search for genes involved in SI; however, some characteristics are also typical for genes termed S-like RNases. The latter are understudied, but have been implicated in various processes. Our results provide incipient hints to the function of S-RNase candidate genes in beets and offer a valuable perspective on S-and S-like RNases in an otherwise understudied group of plants.

## Conclusions

We present evidence that T2/S-RNase genes derived from an ancestral and highly conserved family are present and active in the *Beta* genus. Multiple lines of evidence support the hypothesis that beets contain an S-locus involved in reproductive function. In this work, we were inspired by other genomics-first approaches for S-locus identification, which are especially attractive for studying SI in species that are technically challenging to phenotype–such as wind-pollinated taxa, with inconspicuous flowers. S-locus regions are notoriously difficult to characterize due to their high heterozygosity, extensive polymorphism and an abundance of repetitive elements. The *Beta* candidate S-locus was accordingly challenging to delineate: despite the availability of high-quality resources, we were unable to confidently assert the number of gene copies in the region and found uncertainty in read mapping and annotation. The *Beta* candidate S-region is unique among previously described RSI species; yet, it is as cryptic and complex. This work contributes a groundwork toward elucidating SI in sugar beet and closely related species. Future research will benefit from a combination of transcriptomic approaches and phenotyping in an informed experimental design. Ultimately, a systematic in vivo design will be required to elucidate the SI mechanism in this group.

## Supplementary Information

Below is the link to the electronic supplementary material.

Supplementary file 1: Supporting Data S1 (TXT 255 KB)

Supplementary file 2: Supporting Data S2 (TXT 88 KB)

Supplementary file 3: Supplementary Figures S1–S9 (PDF 1764 KB)

Supplementary file 4: Supplementary Tables S1–S5 (XLSX 62 KB)

## Data Availability

All data supporting the findings of this study are available within the paper and its Supplementary Information.
