# Supplementary material for: Identification of a candidate self-incompatibility locus in beet (Beta vulgaris)
Source: Theor Appl Genet. 2026 Sep 23;139(10):279. doi: 10.1007/s00122-026-05368-6 (PMC13601201; doi:10.1007/s00122-026-05368-6)
Supplement: Supplementary file 1 [file 122_2026_5368_MOESM3_ESM.pdf]

Identification of a candidate self-incompatibility locus in beet (*Beta vulgaris*)

Gil Yardeni, Thomas Holzweber, Juliane C. Dohm, Heinz Himmelbauer

Institute of Computational Biology, Department of Biotechnology and Food Science, BOKU University, Muthgasse 18, 1190 Vienna, Austria

Supplementary Information

Supplementary Figures including legends, Supplementary Table legends, Supporting data legends

Supplementary Figures

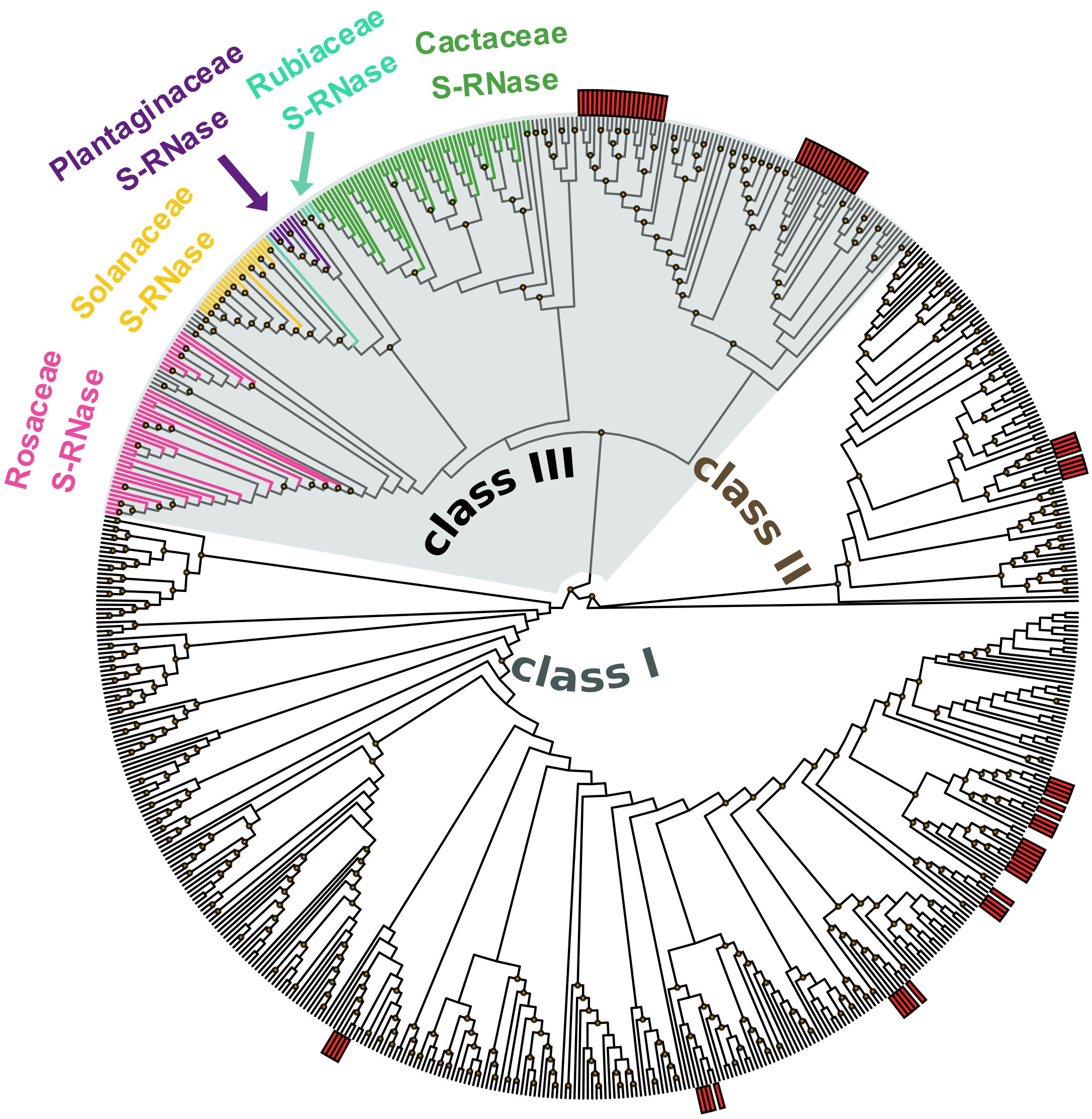

Figure S1. Maximum-likelihood phylogeny of protein sequences of T2/S-RNases, including S-RNase candidate genes identified in four sugar beet genome assemblies. Branch lengths are not to scale but were modified to improve the topology view. Nodes with bootstrap support >95% are marked by a circle. Subtrees corresponding to the three T2/S-RNase classes I, II and III are indicated. Known S-RNases are labeled with the name of the corresponding plant family. Red bars indicate T2/S-RNases identified in sugar beet.

Supplementary Fig. S2 is available on figshare, DOI 10.6084/m9.figshare.33394198

Figure S2. Maximum-likelihood phylogeny of protein sequences of T2/S-RNases as displayed in Figure S1, but including gene names. See legend of Figure S1 for details.

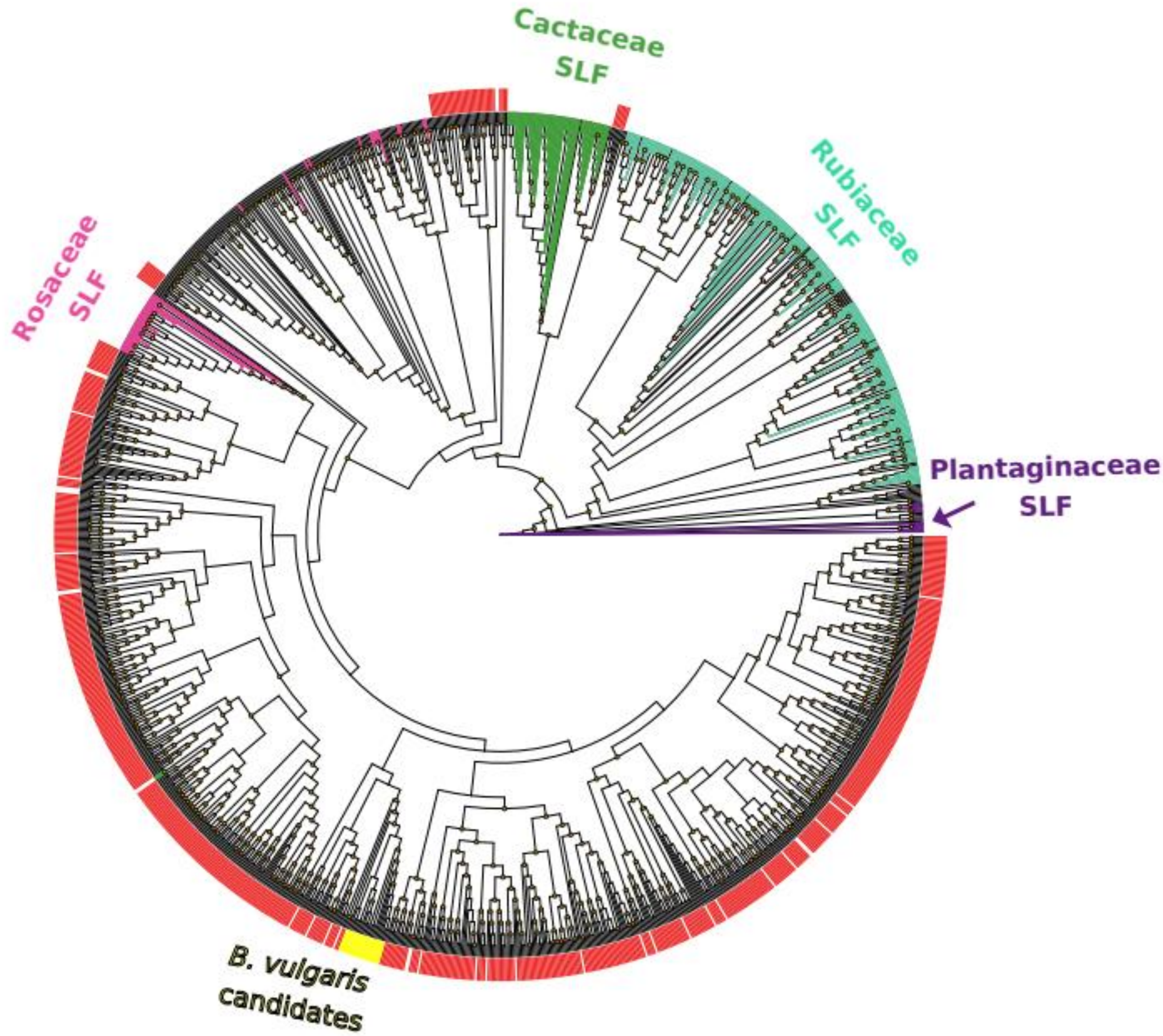

Figure S3. Phylogeny of known F-box SLF/FBA proteins, including F-box candidate genes identified in sugar beet genome assemblies. Branch lengths not to scale, but were modified to improve the topology view. Nodes with bootstrap support >95% are marked by a circle. Known SLF genes are indicated with the name of the taxon. Red bars indicate all sugar beet F-box candidate genes; yellow indicates the F-box genes assigned to the putative beet S-locus.

Supplementary Fig. S4 is available on figshare, DOI 10.6084/m9.figshare.33394282

Figure S4. SLF/FBA phylogeny as described in Figure S3, gene names included. See legend of Figure S3 for details.

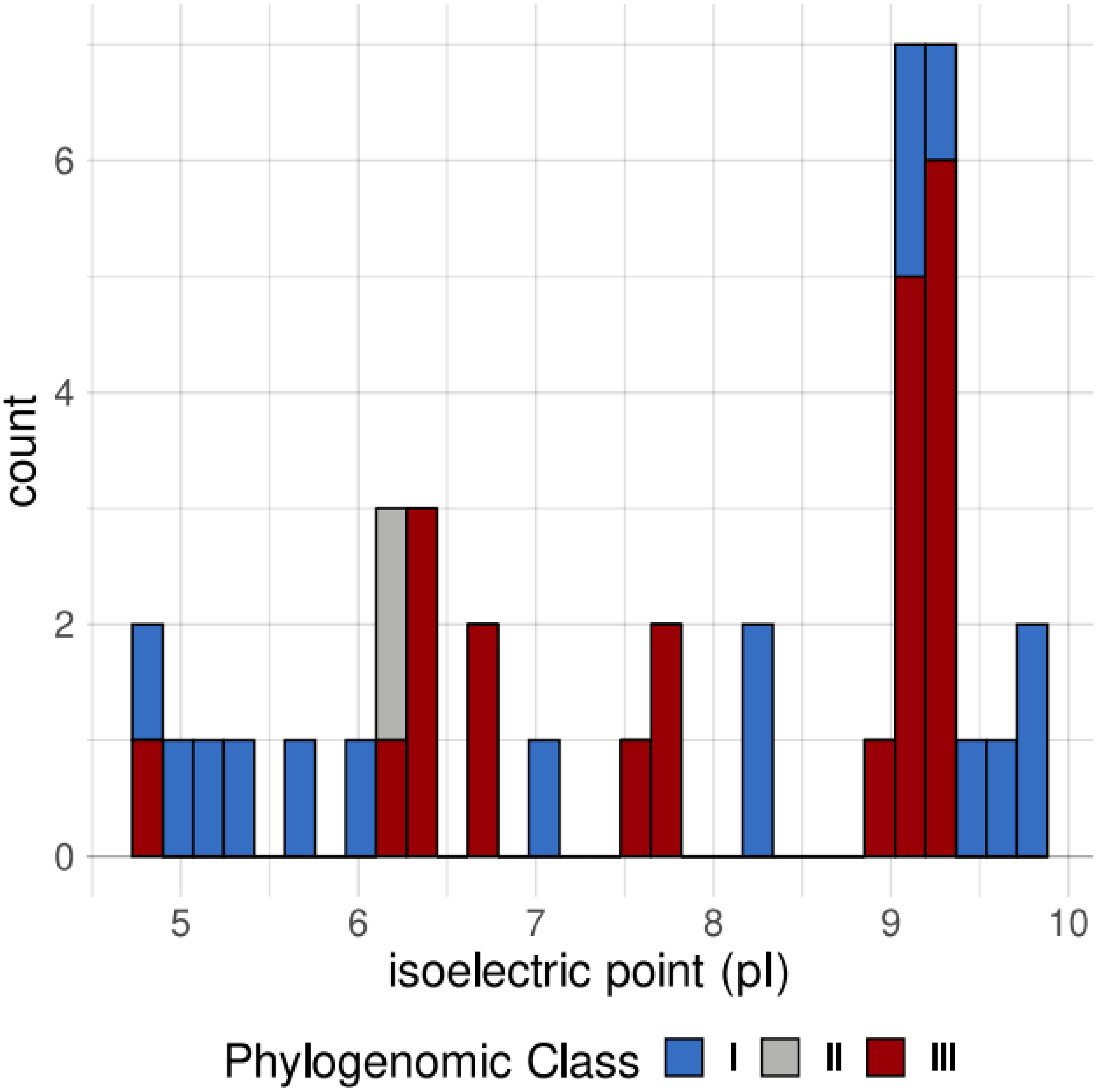

Figure S5. Isoelectric point (pI) of sugar beet T2/S-RNase genes. Classes correspond to topological placement on a T2/S-RNase phylogeny (see Figure S1).

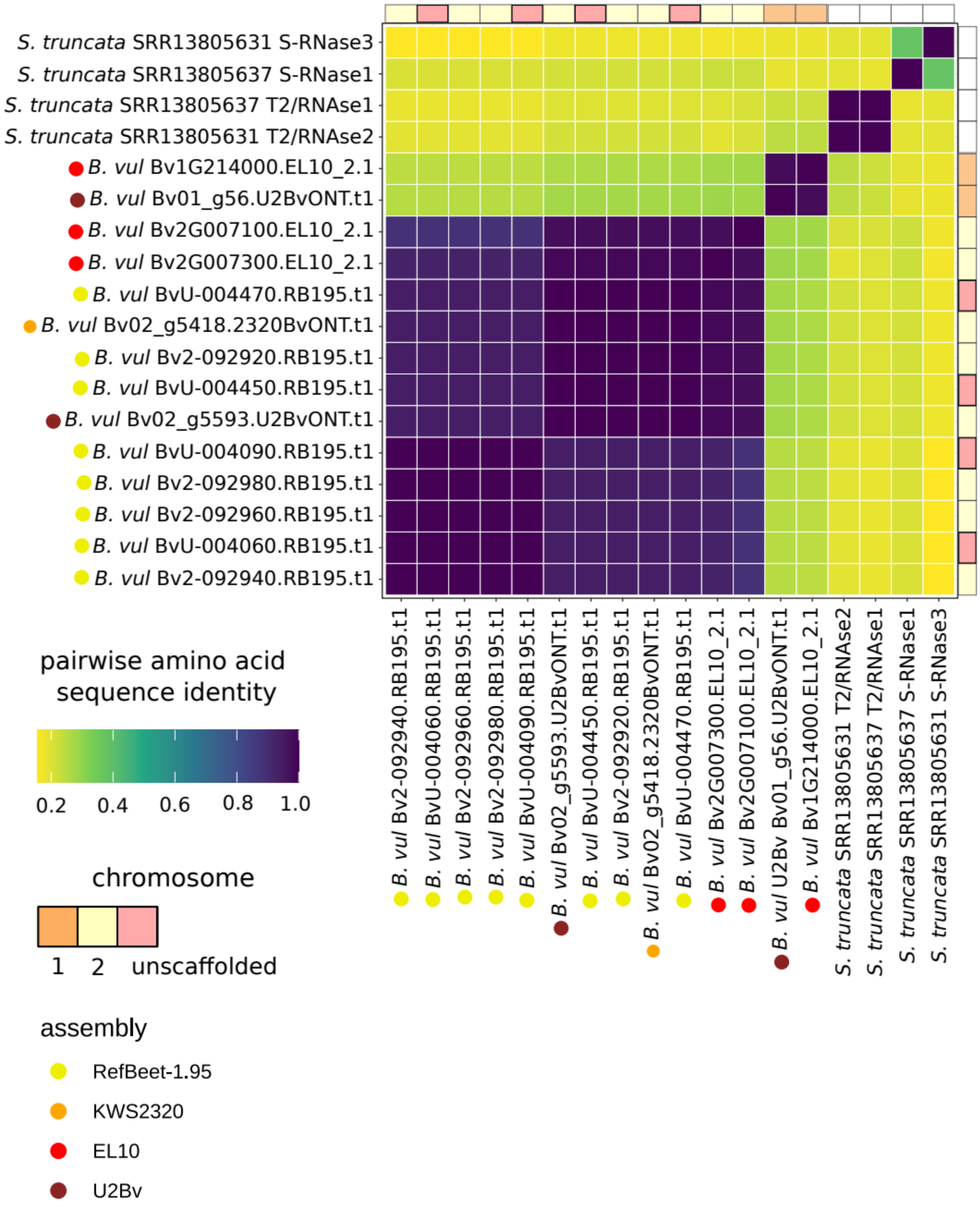

Figure S6. Heatmap of pairwise protein sequence identity among putative S-RNases in sugar beet and representative S-RNase and non-S T2/S-RNase of *Schlumbergera truncata*. Colored circles next to beet gene names indicate source assembly, whereby KWS2320 corresponds to assembly 2320BvONT. Colored bars on top and right indicate the chromosomal assignment of beet genes.

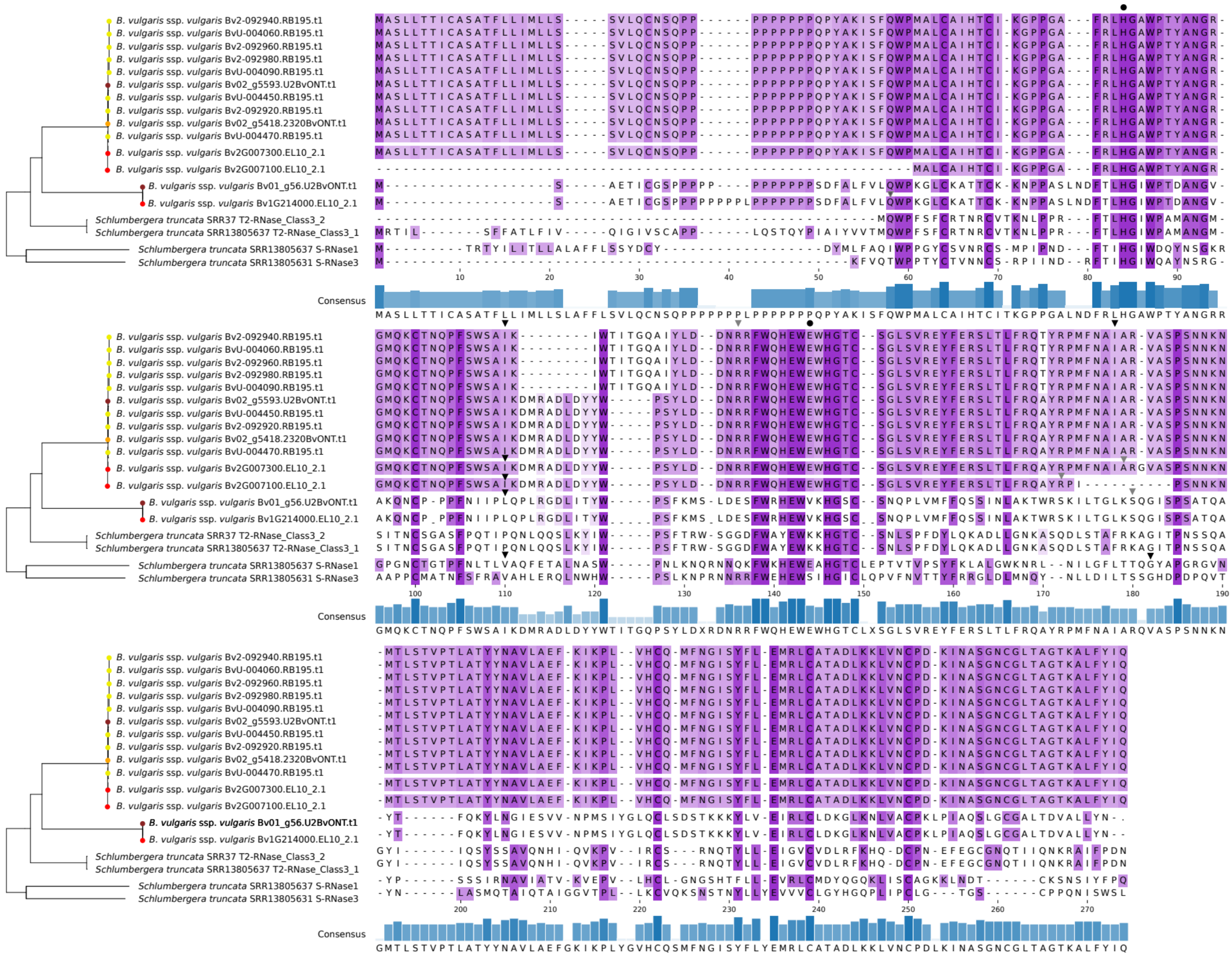

Figure S7. Multiple sequence alignment of sugar beet S-RNase candidate genes, together with S-RNase and non-S T2/S-RNase genes from *Schlumbergera truncata*. A phylogeny on the left represents the relationship inferred between the genes, colored circles next to gene names indicate source assembly of the sequence (see Figure 2). Conserved intron positions are marked with black triangles, grey triangles mark introns that are not conserved. Conserved histidine residues that are essential for RNase activity are marked with black circles.

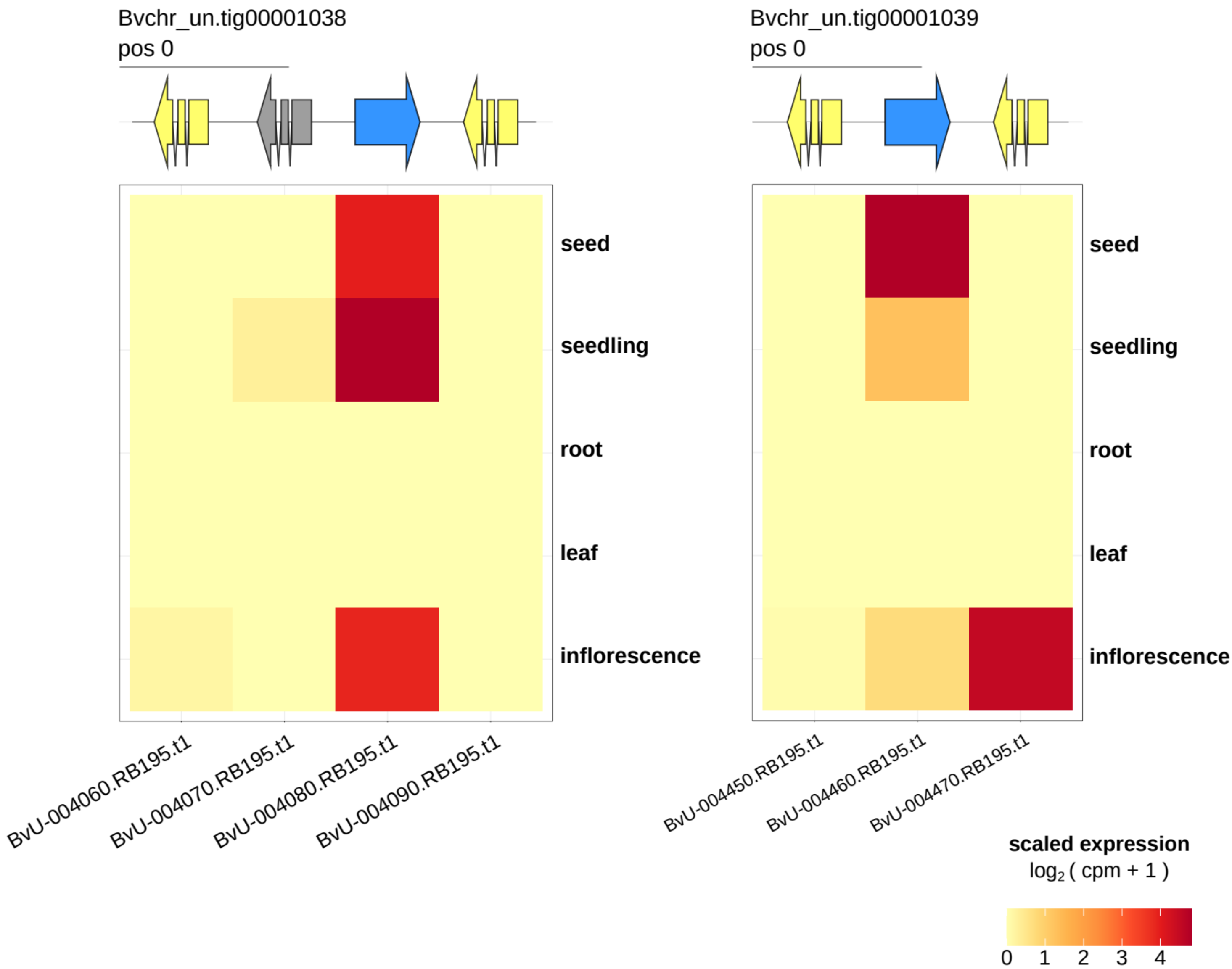

Figure S8. Gene structure and gene expression in two unscaffolded RefBeet contigs. Genes in yellow color: putative S-RNases; blue: F-box genes; grey: gene annotated as a carbon-nitrogen hydrolase. For gene expression in five different tissues, median normalised transformed expression values were calculated.

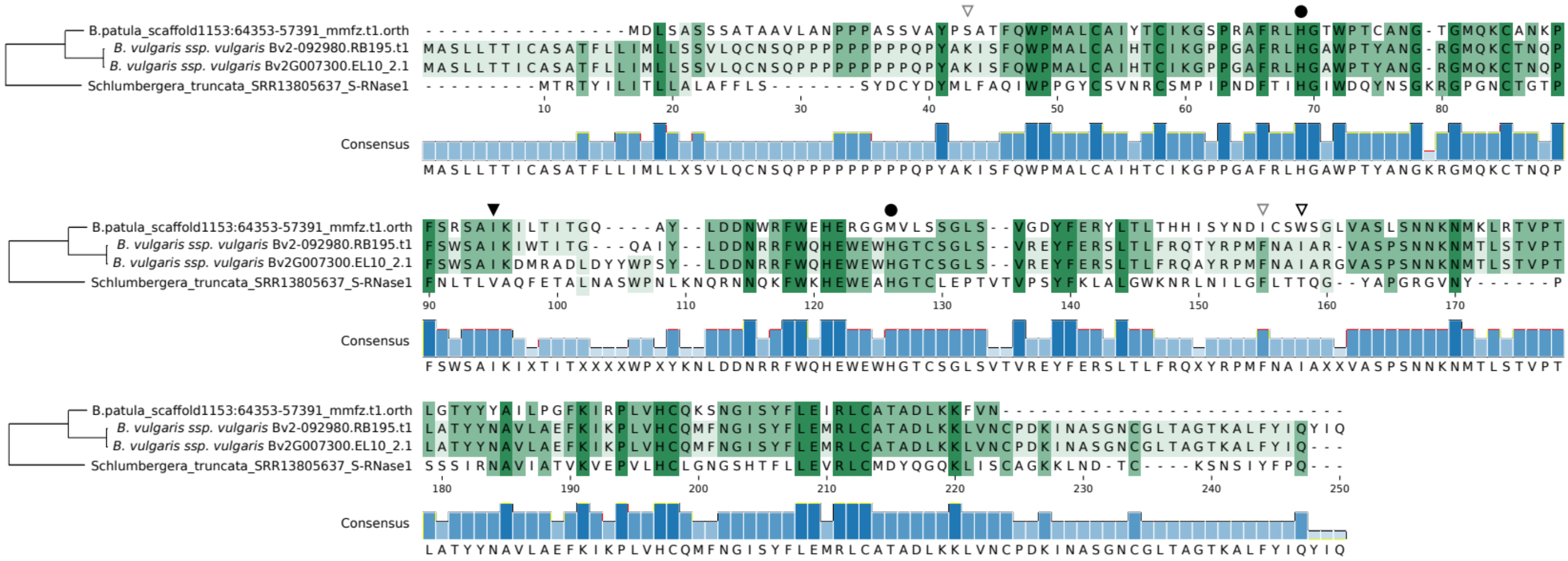

Figure S9. Multiple sequence alignment of S-RNase candidate genes of sugar beet, *Beta patula* and *Schlumbergera truncata*. Intron positions are marked with triangles, where a grey outline indicates introns unique to *B. patula*, black outline indicates introns conserved between sugar beet and *S. truncata*. Black triangles indicate intron positions conserved in all three species. Conserved histidine residues, essential for RNase activity, are marked with black circles.

Supplementary tables

Table S1. RNA data used for transcript assembly and expression quantification, including source information from NCBI.

Table S2. Gene list for the putative S-locus on beet chromosome 2 and flanking region in four sugar beet genome assemblies, according to the original annotation.

Table S3. T2/S-RNase genes identified in the original gene sets of four sugar beet genome assemblies, including chromosomal position, gene properties and functional annotation.

Table S4. SLF candidate genes identified in the original gene sets of four sugar beet genome assemblies, including chromosomal position, gene properties and functional annotation.

Table S5. Gene list for the putative S-locus on beet chromosome 2 and flanking region in four sugar beet genome assemblies, as identified by mapping of cDNA sequences from RefBeet-1.95 to each genome assembly.

Supporting data

Data S1. Nucleotide sequences of RefBeet-1.95 genes, including T2/S-RNases, F-box genes, and anchor genes flanking the S-locus on beet chromosome 2.

Data S2. Protein sequences of RefBeet-1.95 genes, including T2/S-RNases, F-box genes, and anchor genes flanking the S-locus on beet chromosome 2.
